# Impact of Novel Foods on the Human Gut Microbiome: Current Status

**DOI:** 10.3390/microorganisms12091750

**Published:** 2024-08-23

**Authors:** Ailín Martínez, Lidiana Velázquez, Rommy Díaz, Rodrigo Huaiquipán, Isabela Pérez, Alex Muñoz, Marcos Valdés, Néstor Sepúlveda, Erwin Paz, John Quiñones

**Affiliations:** 1 Doctoral Program in Science Major in Applied Cellular and Molecular Biology, Universidad de La Frontera, Av. Francisco Salazar 01145, Temuco 4800000, Chile; a.martinez26@ufromail.cl; 2 Meat Quality Innovation and Technology Centre (CTI-Carne), Universidad de La Frontera, Temuco 4780000, Chile; lidivm15@gmail.com (L.V.); rommy.diaz@ufrontera.cl (R.D.); r.huaiquipan01@ufromail.cl (R.H.); pereznunezisabela@gmail.com (I.P.); a.munoz45@ufromail.cl (A.M.); m.valdes09@ufromail.cl (M.V.); 3 Faculty of Agricultural and Environmental Sciences, Universidad de La Frontera, Av. Francisco Salazar 01145, Temuco 4780000, Chile; nestor.sepulveda@ufrontera.cl; 4 Doctoral Program in Agrifood and Environment Sciences, Universidad de La Frontera, Temuco 4780000, Chile; 5 UWA Institute of Agriculture, The University of Western Australia, Perth 6009, Australia; erwin.pazmunoz@uwa.edu.au

**Keywords:** diet, nutrients, digestive health, gut microbiome, microbial diversity

## Abstract

The microbiome is a complex ecosystem of microorganisms that inhabit a specific environment. It plays a significant role in human health, from food digestion to immune system strengthening. The “Novel Foods” refer to foods or ingredients that have not been consumed by humans in the European Union before 1997. Currently, there is growing interest in understanding how “Novel Foods” affect the microbiome and human health. The aim of this review was to assess the effects of “Novel Foods” on the human gut microbiome. Research was conducted using scientific databases, focusing on the literature published since 2000, with an emphasis on the past decade. In general, the benefits derived from this type of diet are due to the interaction between polyphenols, oligosaccharides, prebiotics, probiotics, fibre content, and the gut microbiome, which selectively promotes specific microbial species and increases microbial diversity. More research is being conducted on the consumption of novel foods to demonstrate how they affect the microbiome and, thus, human health. Consumption of novel foods with health-promoting properties should be further explored to maintain the diversity and functionality of the gut microbiome as a potential tool to prevent the onset and progression of chronic diseases.

## 1. Introduction

In recent years, the microbiome has emerged as a major field of research in biological sciences. The most widely accepted definition of this term, coined by Nobel laureate Joshua Lederberg, is described in a biological context as a community of commensal, symbiotic, and pathogenic microorganisms within a body space or other environment [1]. In 2022, this definition expanded by stating that the microbiome comprises a specific community of bacteria, fungi, archaea, protists, and viruses inhabiting within or on an organism (be it a plant or animal), interacting with each other and their respective environments, leading to the formation of specific ecological niches. This dynamic and interactive system undergoes changes over time and at different scales and is intertwined with macro-ecosystems, including eukaryotic hosts, which play a crucial role in its functioning and health [2].

The microbiome is present in a wide variety of ecosystems, including animals, plants, soils, food, and humans. These microbial communities differ in composition and function both spatially and temporally [2]. Different studies have highlighted the importance of the microbiome and its relationship to fundamental processes, such as food management, metabolism, regulation of the immune system, and prevention of chronic diseases [3,4,5,6,7]. Furthermore, alterations in the microbiome have been shown to be intricately linked to the onset and progression of chronic diseases, such as obesity, type II diabetes, inflammatory bowel diseases, and cardiovascular diseases [8,9].

Research has indicated that the human microbiome plays a critical role in the maintenance of overall health and well-being [10,11]. Although traditionally studied in the context of health and disease, there has recently been a growing interest in understanding how novel foods affect the microbiome and thus human health [12]. This attention is reflected in the inclusion of the microbiome as a crucial component of the European Commission’s Food Strategy 2030 [13]. This perspective has the potential to generate positive impacts on primary food production by promoting sustainable agriculture, strengthening food science, improving human health, and optimising waste management. Diet plays a fundamental role in shaping the composition and function of the microbiome [14].

According to the *Codex Alimentarius*, novel foods denote those that have not been widely consumed either because they have recently emerged into the global retail space thanks to technological innovations, or because their consumption has been historically restricted to specific populations or regions in the world. Such foods are also considered ‘new’ within the framework of existing Codex standards. New food production systems reflect innovations or advancements in pre-existing food technologies that help to produce some of the new foods under discussion [15]. This concept includes foods produced using innovative technologies, such as ultrasound-assisted extraction [16], as well as those derived from new sources and substances from agricultural products, such as chia seeds or noni fruit juice. Additionally, food ingredients from microorganisms, fungi, algae, and insects are novel foods [17]. New raw materials and emerging technologies that extend the shelf life of foods have promoted the development of novel foods [18]. The Novel Food Guide of the European Food Safety Authority (EFSA) classifies these foods into individual substances, simple mixtures, complex mixtures, and whole foods [19], thus providing a structure for their evaluation and regulation. Different definitions have been reported globally (Table 1).

Globally, there is increasing acceptance of novel foods by both the food industry and consumers. Awareness of the relationship between food and health has created a market for novel health-promoting products. These foods may have reduced sugar, salt, or fat content or increased protein content, or may be functional foods to which health-promoting ingredients have been added or harmful ingredients have been removed [18]. Numerous studies have incorporated antimicrobial and antioxidant substances, prebiotics, or other nutrients into edible matrices to prolong shelf life and/or increase the nutritional value of the final packaged food [33,34,35,36,37].

The impact of novel foods on the microbiome has become an increasingly important research area. Different authors have investigated the role of diet in the composition and function of the gut microbiome in adults [3,38,39,40,41]. Diet influences the colonisation, proliferation, or decline of certain microbial strains throughout life [28]. Research has confirmed that the nutrient and fibre content of fruits directly affects the gut microbiome of infants and adults [42,43]. The high presence of polyphenols, oligosaccharides, and fibre, compounds found in a diet rich in fruits and vegetables, has been associated with a reduced risk of chronic diseases. Numerous benefits derived from this type of diet are due to the interaction between the aforementioned components and the gut microbiome, which selectively promotes specific microbial species and increases microbial diversity [42,44].

In contrast, prebiotic and probiotic foods are known to positively influence the microbiome by promoting the growth of beneficial bacteria and improving the digestive function [45]. Furthermore, fermented foods, in addition to being an ancient technique for food preservation, have favourable effects on health by modulating the microbiome. These foods provide nutrients that stimulate or inhibit the growth of specific members of the gut microbiome, establishing colonisation in the gut and/or interacting with the resident microbiota [46]. A recent study evaluated the effect of the overall intake of fermented plants on microbial and metabolomic differences between consumers and non-consumers over four weeks. The authors observed that the diversity of microorganisms was significantly different between consumers and non-consumers. It was found that the microorganisms associated with people consuming fermented foods included those typically present in fermented foods, such as *Lactobacillus acidophilus*, *Levilactobacillus brevis*, *Lactobacillus kefiranofaciens*, *Lentilactobacillus parabuchneri*, *Lactobacillus helveticus*, and *Latilactobacillus*, as well as those unrelated to them, including *Streptococcus disgalactiae*, *Prevotella melaninogenica*, *Enorma massiliensis*, *Prevotella multiformis*, *Enterococcus cecorum*, and *Bacteroides paurosaccharolyticus* [47].

Currently, there are diverse new foods, nutrients, products, nutritional supplements, medicines, and eating habits. In this context, the question arises of whether these changes may have an impact on the microbiome and, consequently, on oral, stomach and gut health. Therefore, the aim of this review was to assess the effects of novel foods on the human gut microbiome.

## 2. Methodology

The revision was carried out using the scientific databases Scielo, PubMed, Google Scholar, Web of Science, and ScienceDirect, considering the literature published since 2000 and paying special attention to the last decade (2014–2024).

## 3. Microbiome

### 3.1. Microbiome Definition

Researchers have focused on understanding the characteristics of the human microbiome since the inception of the National Institutes of Health-funded Human Microbiome Project in 2007 [48]. The microbiome is the totality of organisms and their genetic make-up [49]. In this context, several authors have conceptualised the microbiome as a complex community of microorganisms that influence host health and evolution [11,50,51,52] (Table 2).

Microbiome studies span a wide range of disciplines, highlighting the important roles of microbial communities in diverse ecosystems and hosts. This field of research is a focal point in areas such as biology, biomedicine, ecology, evolution, bioinformatics, and statistics [49,58,59,60,61]. The influence of the microbiome on host evolution, heritability, and phenotypic effects highlights the complex interaction between hosts and their microbiome. The human microbiome is composed of many microbial cells, exceeding the number of cells in the human body. In addition, the human microbiome is estimated to contain a substantial amount of genetic material, with a much larger number of genes than the human genome (100-fold) [62].

Several factors affect the human microbiome, including diet, intensity of physical activity, and microbial interactions, which can modify its composition and diversity [63,64]. In a recent study, Gasesa et al. [64] assessed the gut microbiomes of 8208 Dutch individuals and analysed bacterial composition, function, antibiotic resistance, and virulence factors. Their findings revealed that environment and cohabitation play a predominant role in modulating the microbiome, highlighting that approximately 6.6% of bacterial taxa are heritable, whereas approximately 48.6% of taxonomic variation is significantly influenced by cohabitation. In addition, they identified 2856 associations between the microbiome and health, noting that unrelated diseases share a common microbial signature that is not influenced by comorbidities. They also found 7519 associations between microbiome characteristics and various aspects such as diet, socioeconomic status, and environmental factors in early and current life.

### 3.2. Microbiome Characterisation

**Figure 1:**
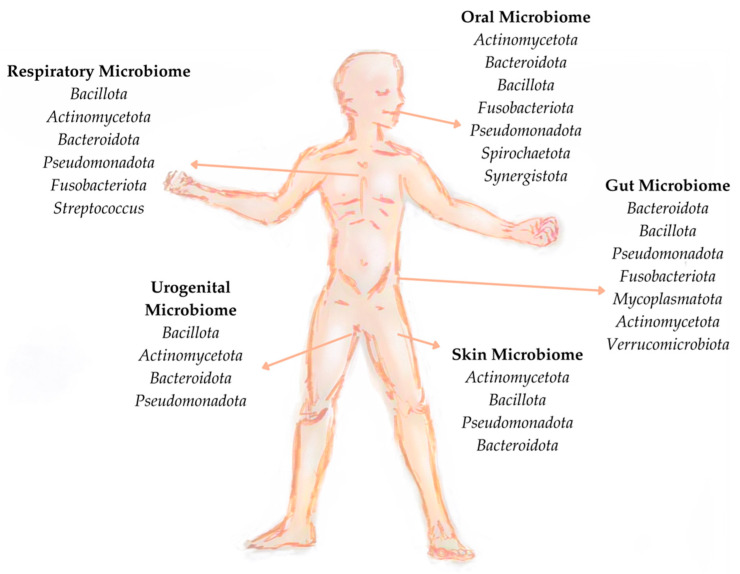
Main diversity of the human microbiome.

Microbiological research has progressed from van Leeuwenhoek’s discoveries to the current metagenomic approaches. Studies such as the Human Microbiome Project and Metagenomics of the Human Intestinal Tract (MetaHIT) have contributed to our knowledge of the microbial diversity in healthy individuals [65]. A healthy human microbiome is composed of more than thirty trillion microorganisms per person, which include bacteria, viruses (bacteriophages and human viruses), and yeasts [66]. These microorganisms inhabit various parts of the human body, such as the skin, oral cavity, and gastrointestinal, respiratory, and genitourinary tracts (Figure 1). Together, they account for 1–3% of a person’s total body weight. Microbial composition varies significantly along the gastrointestinal tract, with differences in the main genera present in each region [67].

In humans, the gut microbiome has been described as established within the first three years of life [68]. Several factors influence the gut microbiome, including birth, lactation, diet, susceptibility, genetics, and antibiotic use. The gut microbiome during the developmental stage of an infant is formed and modulated by a combination of microbiota and oligosaccharides present in breast milk [69]. Breast milk consists of a complex microbial community containing commensal, mutualistic, or potentially viable probiotic microbiota capable of developing the oral and intestinal microbiomes of lactating infants [70]. Moreover, the composition, diversity and dynamics of the infant gut microbiome are influenced by different milk components, such as fat globules, oligosaccharides, and exosomes, as well as lactation type [71].

The gut microbiome is the largest microbial community in the human body [72], comprising approximately fifty microbial phyla [73]. *Bacteroidota* and *Bacillota* are the dominant phyla in the healthy human gut. Additionally, *Pseudomonadota* and *Fir* were found in abundance, whereas the genera *Bifidobacterium*, *Escherichia*, *Clostridium*, and *Akkermansia* were also detected at lower levels [74,75]. *Methanobacteriales* and *Methanomassiliicoccales* are the two classes of *Archaea* that predominate the intestinal mycobiome [76]. In the case of fungi, the phylum *Ascomycota* is the most representative, followed by *Basidiomycota* and *Mucoromycota* [77,78]. *Caudovirales*, *Myoviridae*, *Siphoviridae*, and *Circoviruses* dominate the intestinal virome [79,80]. A rich and diverse microbial community contributes to a healthy and balanced gut microbiota composition [81].

A recent study by Park et al. [71] investigated microbial colonisation in intrauterine environments and in the gastrointestinal tracts of foetuses. In this study, 19,597,239 bacterial sequences were identified in 641 samples obtained from 141 pregnant women and 178 newborns. They observed a distinct bacterial composition in the meconium of newborns, supporting the idea that microbial colonisation begins during normal pregnancy. They also found that the microbiome in neonatal gastric fluid was similar, but not identical, to that of maternal amniotic fluid. These findings are consistent with the theory that foetuses ingest amniotic fluid in utero and that their urine returns to the fluid under normal physiological conditions. Qin et al. [82] performed 16S rRNA gene sequencing of midstream urine samples from 1172 healthy middle-aged and elderly individuals. The basic microbiota included six dominant genera (mean relative abundance > 5%), *Prevotella*, *Streptococcus*, *Lactobacillus*, *Gardnerella*, *Escherichia-Shigella*, and *Veillonella*, and 131 low-abundance genera (0.01–5%). The composition and diversity of the genitourinary microbiome differed according to sex and fluctuated with age. The prevalence of urotypes in the male gender also harboured *Acinetobacter*, *Corynebacterium*, *Staphylococcus* or *Sphingomonas*, *Peptoniphilus*, *Ezakiella*, and *Porphyromonas*.

Gut microbiome research has opened new avenues for exploring the impact of microbiota on other physiological systems, including the skin. The skin surface harbours a complex and diverse microbial ecosystem that interacts with the immune system and affects dermatological health. Several key roles of the skin microbiome have been identified, including nutrient synthesis, pathogen growth suppression, immune response modulation, and epidermal differentiation regulation. The skin microbiota is composed of both stable residents and transient colonisers [83]. Most (>90%) of the bacteria of the human skin microbiome are classified into four types: *Actinomycetota* (52%), *Bacillota* (24%), *Psudomonadota* (16%), and *Bacteroidota* (6%) [84].

The diversity of the human oral microbiome has also documented in the expanded Human Oral Microbiome Database (eHOMD), which comprises 775 microbial species, of which 57% are officially identified, 13% are culturable but not yet named, and 30% are unculturable phylotypes [85]. This database also hosts 2074 oral/nasal genomes, covering 529 taxa, including *Actinomycetota*, *Bacteroidota*, *Bacillota*, *Fusobacteriota*, *Pseudomonadota*, *Spirochaetota*, and *Synergistota* among the most representative bacterial communities [86]. In addition, eHOMD includes genomes of a new phylum of nano-microorganisms, known as “candidate *phyla* radiation”, of bacterial organisms including 35 phyla and consists of more than 15% of the domain Bacteria [87]. Multiple factors, such as host genetics, maternal transmission, and environmental factors, such as dietary habits, oral hygiene practices, medications, and systemic factors, influence the composition of the oral microbiome [88]. A study in North American populations identified that the phyla *Bacillota*, *Pseudomonadota*, *Bacteroidota*, and *Actinomycetota*, and the genera *Streptococcus* spp., *Haemophilus* spp., *Neisseria* spp., *Prevotella* spp., *Veillonella* spp., and *Rothia* spp. were the most prevalent [89].

Recent studies on the respiratory microbiome have revealed its importance in various pulmonary infections. The healthy nasal cavity is inhabited by microbial species, such as *Corynebacterium* spp., *Dolosigranulum* spp., and *Moraxella* spp. [90]. The oropharyngeal microbiome is mostly composed of *Streptococcus* spp. and has higher microbial diversity than the nasopharyngeal microbiome [91]. Disruption of microbial balance in the upper airways contributes to inflammation and obstruction of the lower airways. Lung diseases, such as asthma, cystic fibrosis, chronic obstructive pulmonary disease, and idiopathic pulmonary fibrosis, have been shown to be associated with changes in the diversity and abundance of specific bacterial taxa [92]. The microbiota of the upper respiratory tract has also received attention during the COVID-19 pandemic because of its role in viral infections [93].

### 3.3. Factors Affecting Microbiome Composition

The microbiome properties are determined by a combination of external and internal factors. These factors include aspects of host physiology, genetics, and the environment, which contribute to the observed variations in the gut microbiome between individuals [72]. Kurilshikov et al. [94] studied the effect of host genetics on the composition of the gut microbiome in 18,340 individuals from twenty-four population cohorts of European, Hispanic, Middle Eastern, Asian, and African descent. They identified thirty-one loci associated with bacterial abundance in the gastric microbiome, including the lactase gene locus (LCT), which showed an age-dependent association with *Bifidobacterium* abundance. Furthermore, they found that the LCT locus is associated with multiple diet- and metabolism-related phenotypes and that the causal effect of this locus was confirmed in 49 of 51 phenotypes evaluated. Likewise, these authors suggested that genetic variants affecting bacterial abundance in the gastric microbiome participate in the regulation of metabolism and have causal effects in ulcerative colitis and rheumatoid arthritis.

Age, sex, diet, smoking, physical activity, stress, temperature, medications, diseases, and bacteriophages all contribute to short- and long-term changes in the microbiome [60]. Nutrition [73], antibiotics [95], mode of delivery, and cessation of breastfeeding [96] have also been shown to have a major impact on the gut microbiome in adults. In addition, environmental exposure to contaminants and biological agents shapes the gut microbiota and has an impact on the overall health status [97]. In addition, lifestyle factors, such as reduced physical activity and poor diet, have been associated with changes in the abundance and composition of the gut microbiome, which influence the development of diseases such as obesity [98]. Elevated maternal psychosocial stress has also been associated with alterations in the infant gut microbiome, affecting the diversity and levels of health-promoting bacteria, such as *Lactobacillus gasseri* and *Bifidobacterium pseudocatenulatum* [99]. Zhang et al. [100] suggested that stress can lead to dysbiosis of the gut microbiota, contributing to the development of affective disorders. In addition, exposure to stress in early life has been associated with changes in the human gut microbiome with implications for mental health [99].

A crucial factor that affects the composition of the human gut microbiome is diet [97]. Su and Liu [101] suggested that different food components, such as carbohydrates, fats, proteins, minerals, vitamins, food additives, cooking, and processing can alter the structure and function of the gut microbiome. Long-term unhealthy dietary habits are a key factor in the development of non-communicable diseases. Negative effects on the gut microbiome and host due to dietary fibre deficiency have been described, especially in people with obesity or metabolic diseases [102]. Additionally, the oral microbiome can influence the composition of the gut microbiome. Under certain circumstances, such as oral–intestinal barrier dysfunction, oral bacteria can translocate to the gut [103]. Hu et al. [104] found that *Streptococcus salivarius* was present in both the oral cavity and gut of individuals with Crohn’s disease, suggesting that oral bacteria can colonise the gut and potentially influence the composition of its microbiome.

## 4. Foods and Microbiome

**Figure 2:**
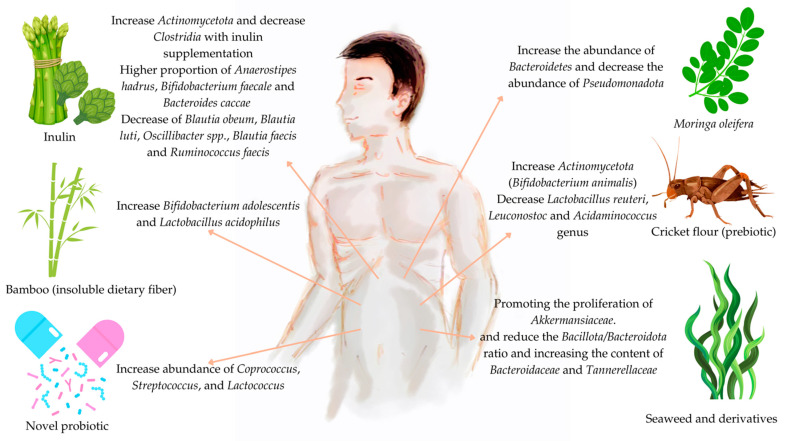
Impact of some novel foods on the human gut microbiome.

Food diversity has a significant effect on the gut microbiome by influencing its overall composition and structure. The interaction between food components and gut microbiota occurs through various mechanisms, such as nutrient competition, antagonism, and support for the stability of the microbiota, which have an impact on its richness and diversity [105]. Prebiotic fibres, polyphenols, and polyunsaturated fatty acids play essential roles in shaping the gut microbiome [106]. Studies have shown that diets based on prebiotics and fibre stimulate the proliferation of *Faecalibacterium* and *Bacteroides* [105,107]. In contrast, diets rich in animal fat have been associated with an increase in pathogenic species, such as *Lachnoclostridium* [108]. Similarly, increased consumption of refined cereals and gluten has been correlated with decreased microbial diversity, which has implications for intestinal health and susceptibility to diseases [106,109]. The response of the gut microbiome to foods can be affected by dietary selection pressures throughout life, especially during the initial stages of life when food preferences are established [110]. Therefore, the consumption of novel foods may affect the maturation of the human gut microbiome by influencing its composition and diversity (Figure 2).

### 4.1. Nutrients

#### 4.1.1. Carbohydrates

Carbohydrates are classified into digestible and non-digestible substrates. Digestible carbohydrates such as galactose, glucose, and fructose are enzymatically broken down in the small intestine and absorbed as glucose into the bloodstream [111]. In contrast, non-digestible carbohydrates, also known as dietary fibre, are resistant to digestion in the small intestine and reach the large intestine. These include lignin, resistant starches, non-digestible oligosaccharides, and non-starch polysaccharides [112,113]. Dietary fibres can be classified according to their water solubility (soluble or insoluble) or their fermentability in the colon (fermentable or non-fermentable). Non-fermentable dietary fibres, such as cellulose, hemicellulose, lignin, and resistant starch, are insoluble [114]. These insoluble dietary fibres are not digestible by human digestive enzymes; they reach the large intestine intact, where members of the gut microbiota metabolise them [115]. Vitaglione et al. [116] proposed that insoluble dietary fibre intake increased the relative abundance of beneficial gut bacteria and reduced harmful bacteria. In contrast, fermentable dietary fibres, such as inulin, pectin, beta-glucan, fructo-oligosaccharides, and galacto-oligosaccharides, are hy-soluble [111]. Soluble dietary fibre comprises wheat dextrin, pectin, gums, β-glucan, psyllium, and fructans, as well as part of hemicellulose [117]. They are derived from cereals, fruits, vegetables, and legumes [118].

In addition, dietary fibres that are resistant to digestion by the host are called microbiota-accessible carbohydrates [119]. Dietary fibre is a key nutrient for maintaining the diversity of the gut microbiota [3]. Short-chain fatty acids are metabolites produced by fermentation of dietary fibre. Steimle et al. [120] used a gnotobiotic mouse model containing a synthetic human gut microbiome composed of fourteen strains from five different phyla. They evaluated the effect of concentrated crude dietary fibre concentrates from pea, oat, psyllium, wheat, and apple on the synthetic human gut microbiome. They showed that the abundance of *Eubacterium rectale*, *Roseburia intestinalis*, and *Bacteroides ovatus* increased, whereas the abundance of *Akkermansia muciniphila* decreased.

#### 4.1.2. Proteins

Proteins also significantly affect the composition and function of the human gut microbiome [121]. Animal proteins are present in meat, fish, eggs, and dairy products and are a rich source of essential amino acids. Several studies have suggested that diets rich in animal protein can lead to a decrease in beneficial gut bacteria and an increase in harmful ones [122,123,124]. In this regard, the consumption of red meat and dairy products leads to an increase in the abundance of bile-tolerant anaerobic bacteria, such as *Bacteroides*, *Alistipes*, and *Bilophila* [125]. Wu et al. [121] analysed the bacterial composition of dietary specimens based on the relative abundance of fecal bacteria. They found that pathogenic bacteria were more abundant in omnivorous participants than in vegetarians, whereas probiotic species were more abundant in vegetarians. Detailed dietary assessments further revealed that proteins of plant and animal origin can modulate the relative abundance of the pathobionts *Bilophila* and *Lachnoclostridium*.

Protein quality and processing conditions play crucial roles in gut microbiota; therefore, it is important to select high-quality proteins with positive effects on gut health [123]. Consumption of fish rich in protein and omega-3 fatty acids is associated with an increase in microbiome diversity and the presence of beneficial bacteria such as *Bifidobacteria* and *Lactobacilli*. This increase in microbial diversity is correlated with improved gut health and reduced systemic inflammation [126]. Furthermore, plant-based proteins found in legumes, grains, nuts, and seeds significantly influence the gut microbiome. These protein sources are often rich in fibre, which supports the growth of beneficial bacteria [127]. For example, soy proteins have proven prebiotic effects [128]. The effects of essential amino acids, such as glutamine, on the gut microbiome have also been studied. Glutamine is a key amino acid for gut health, as it serves as an energy source for gut cells and plays a crucial role in maintaining the integrity of the intestinal barrier. As demonstrated by Zambom de Souza et al. [129], glutamine supplementation affects the gut microbiota composition in obese individuals. The authors of that study found that glutamine supplementation decreased the *Bacillota*-to-*Bacteroidota* ratio (from 0.85 to 0.57) and reduced *Actinomycetota* in obese individuals, compared to alanine supplementation.

#### 4.1.3. Fats

Consumption of high-fat foods is associated with a reduction in intestinal bacterial diversity [130,131] and enhances intestinal permeability directly by stimulating proinflammatory signalling cascades and indirectly via increasing barrier-disrupting cytokines (TNFα, interleukin IL) 1B, IL6, and interferon γ (IFNγ) and decreasing barrier-forming cytokines (IL10, IL17, and IL22). A high-fat diet negatively modulates the intestinal mucus composition and enriches the gut microflora with barrier-disrupting species [132,133]. These changes affect the immune system and lead to low-intensity systemic inflammation. Foods high in saturated fats have been associated with unfavourable changes in the gut microbiome. A high intake of fat and saturated fatty acids may negatively affect microbiota richness and diversity, and diets high in monounsaturated fatty acids may decrease total bacterial numbers, whereas dietary polyunsaturated fatty acids had no effect on richness and diversity [131].

A comparison of the gut microbiota in individuals consuming saturated fatty acids (SFAs) above the WHO recommendation, including those with obesity or metabolic disorders, revealed a higher prevalence of the *Anaerotruncus* genus, a butyrate-producing bacteria linked to obesity. Females with high SFA intake exhibited elevated levels of *Campylobacter*, *Flavonifractor*, *Blautia*, and *Erysipelatoclostridium*, while males showed increased abundance of *Eisenbergiella* and *Anaerotruncus*, a *Clostridiales* genus. The microbial richness in women with high SFA consumption is linked to a heightened susceptibility to obesity and related inflammatory conditions [133]. Diets containing high levels of polyunsaturated fatty acids (PUFAs) (soybean oil), monounsaturated (MUFAs) (olive oil), and saturated fatty acids (SFAs) (coconut oil) have the potential to elicit varied effects on the intestinal microbiota diversity, insulin sensitivity, and the metabolic utilisation of fatty acids (including biosynthesis and degradation) in PPARγ knock-out mice, as discussed by López-Salazar et al. [134]. The group consuming a diet rich in PUFAs exhibited the most diverse microbial population, particularly abundant in *Akkermansia muciniphila*, reduced inflammation, and enhanced insulin sensitivity in comparison to the SFA-rich coconut oil group. Conversely, the MUFA cohort displayed responses to gut microbiota and insulin sensitivity that were comparable to those observed in the PUFA group. In contrast, the SFA group, which consumed coconut oil, demonstrated the lowest microbial diversity, a substantial 9-fold elevation in LPS levels, hepatic steatosis, heightened lipogenesis, elevated LDL levels, and diminished fatty acid oxidation.

## 5. Nutritional Supplements

Nutritional supplements, including vitamins, minerals, probiotics, prebiotics, and bioactive compounds are known for their ability to modulate the gut microbiome. Dietary interventions targeting the gut microbiota have shown promise in the management of metabolic disorders such as obesity and type II diabetes. In the post-acute COVID-19 syndrome setting, dietary interventions targeting the microbiota have shown efficacy in alleviating gastrointestinal symptoms, underscoring the therapeutic efficacy of dietary modulation of gut microbiota in improving health outcomes [135].

### 5.1. Vitamins

Certain vitamins when administered in high quantities or targeted at the large intestine, have demonstrated the ability to positively influence the gut microbiome. This is achieved through various mechanisms, including enhancing the population of presumed commensal bacteria such as vitamins A, B2, D, E, and beta-carotene, promoting or preserving microbial diversity (vitamins A, B2, B3, C, K) and richness (vitamin D), boosting the production of short-chain fatty acids (vitamin C), or increasing the prevalence of short-chain fatty acid-producing microorganisms (vitamins B2, E). Conversely, vitamins like A and D play a role in regulating the gut immune response or barrier function, thereby exerting an indirect impact on gastrointestinal health and the microbiome [136]. A study in 306 infants in Bangladesh randomised to receive a single high dose of vitamin A or placebo within 48 h of birth showed that boys receiving vitamin A had a higher abundance of fecal *Bifidobacterium* than boys receiving placebo; however, this difference was not seen in girls. For girls in late infancy, a positive association of plasma retinol with *Actinomycetota* (the phylum containing *Bifidobacterium*) and the commensal *Akkermansia* was found. However, there were no differences seen in the study population overall for *Bifidobacterium* and *Pseudomonadota* [137].

Another study suggested that high-dose vitamin D supplementation alters the microbiome composition of adolescent girls. Fifty girls were supplemented with 50,000 IU of cholecalciferol weekly for nine weeks. In this population, *Bacteroidota* and *Lactobacillus* decreased, whilst *Bacillota* and *Bifidobacterium* increased after supplementation [138]. Increased intake of vitamins B2, B5, B6, and B12 in lactating women was correlated with increased relative abundance of *Prevotella* and decreased relative abundance of *Bacteroides* [139]. On the other hand, in an intervention study, a group received iron supplementation with vitamin E and another group iron. Significantly different changes in the composition of the microbiome were observed over time, particularly in the *Bacteroides* and *Bacillota* phyla. In the group receiving iron plus vitamin E, there was a decrease in *Bacteroides*, an increase in *Bacillota*, and an increase in the abundance of *Roseburia*, compared to the group receiving iron alone [140].

The gut microbial population of Japanese women on a vitamin K-rich diet was assessed using a 3-day food diary. Analysis of the microbiome allowed women to be divided into two groups. One group was characterised by a high relative abundance of *Ruminococcaceae* and *Bacteroides*, and this group had a significantly lower vitamin K intake. The other group had a high relative abundance of *Bifidobacterium* and *Lactobacillus* and a higher vitamin K intake [140]. An observational study showed that vitamin C intake was associated with a decrease in *Bacteroidota* in adults with cystic fibrosis [141]. In addition, a dose of 500 mg/day of vitamin C for 4 weeks resulted in an increase in alpha diversity compared to baseline and placebo [136]. Although these authors did not detect significant changes in bacterial composition measured at the genus or species level, there was an increase in total short-chain fatty acids (SCFA), driven by higher concentrations of butyric and propionic acid after supplementation.

### 5.2. Minerals

Minerals, such as iron, zinc, magnesium, and calcium, are micronutrients that significantly influence the composition and function of the microbiome [41,142]. In vivo studies have shown that zinc deficiency alters the composition of the gut microbiome, with decreased biodiversity, increased inflammatory markers, and impaired functional potential involved in gut–brain signalling [143,144]. On the other hand, high calcium intake has been associated with a higher proportion of *Clostridium* cluster XVIII in men [145], an effect related to the ability of calcium to bind bile acids and reduce toxicity in the gut, creating a more favourable environment for beneficial bacteria. Iron supplementation may reduce *Bifidobacterium* levels and increase *Lactobacillus* levels in children [146]. Phosphorus supplementation has also been reported to increase microbial diversity and short-chain fatty acid levels in feces [147]. In addition, magnesium has shown to have a positive impact on the composition of the gut microbiome as well as on the metabolism of vitamins B1 and D in patients with metabolic syndrome, type II diabetes, and obesity [147,148]. Furthermore, it has been reported that various members of the gut microbial ecosystem can influence the bioavailability of micronutrients by controlling their absorption, particularly phosphorus and calcium [149].

### 5.3. Prebiotics, Probiotics, Synbiotics, and Postbiotics

Prebiotics are non-digestible compounds such as fructo-oligosaccharides, galacto-oligosaccharides, isomalto-oligosaccharides, and xylo-oligosaccharides, which are metabolised by intestinal microorganisms and exert beneficial effects through their selective metabolism in the intestinal tract [149]. The main by-products of bacterial prebiotic metabolism are short-chain fatty acids, such as acetate, butyrate, and propionate [150]. The resulting changes in microbial composition and metabolite concentrations from prebiotic administration influence epithelial, immune, nervous, and endocrine signalling in the host. In addition, they are predictors of health benefits such as improvements in gut function, immune response, glucose and lipid metabolism, bone health, and regulation of appetite and satiety [151]. Prebiotics are found in plants, such as onions, asparagus, garlic, chicory, Jerusalem artichoke, oats, and wheat, and can induce metabolic activities in the colon by stimulating bacterial growth in the gut. Carbohydrates that are accessible to the microbiota act as prebiotics, facilitating their metabolism into short-chain fatty acids [150].

The scientific literature classifies prebiotics as functional foods given their role in health promotion and disease prevention [152]. The main premise of prebiotics is that carbohydrates selectively fermented in the colon modulate the levels of resident *Lactobacillus* and *Bifidobacterium*, thereby promoting health-promoting effects. However, current targets for prebiotics have expanded beyond lactic acid bacteria to encompass a wider range of positively responding microorganisms [151]. These include health-promoting genera, such as *Roseburia*, *Eubacterium* spp., *Akkermansia* spp., *Christensenella* spp., *Propionibacterium* spp., and *Faecalibacterium* spp. Prebiotics can stimulate the growth of these and other bacterial groups directly or indirectly through cross-feeding interactions. These interactions are essential for maintaining a balanced and functional microbiome that contributes significantly to the overall health of the host [153].

Probiotics are among the most studied supplements in relation to the gut microbiome. Traditionally, lactobacilli, bifidobacteria, and other lactic acid-producing bacteria (LAB) isolated from fermented dairy products and the fecal microbiome have been used as probiotics. Fermented foods are the most common natural source of probiotic lactic acid bacteria. Their consumption has been associated with a reduced risk of type II diabetes and cardiovascular disease [154], as well as a beneficial metabolomic profile [34]. These foods have also been described as the main source of LAB in the human intestinal microbiome [155]. Furthermore, it has been shown that probiotics can influence the composition of the microbiome through the modulation of the gene expression of microorganisms in the microbiome. This can be achieved through the administration of probiotics that contain genes encoding proteins that interact with host re-acceptors [156]. Suez et al. [156] demonstrated that probiotics can modify the gut microbiome by increasing the abundance of *Lactobacillus* and *Bifidobacterium*. This effect can be observed in people with antibiotic-induced dysbiosis, where probiotics help restore microbial diversity and improve gut function [157].

Moreover, probiotics have been shown to influence gut microbial composition by preserving alpha diversity and ameliorating changes induced by factors such as antibiotics, promoting the growth of beneficial bacteria such as *Faecalibacterium prausnitzii* [157]. *Akkermansia muciniphila*, *Ruminococcus bromii*, *Faecalibacterium prausnitzii*, *Anaerobutyricum hallii*, and *Roseburia intestinalis* are the five most relevant gut-derived Next Generation Probiotics (NGPs) that have demonstrated therapeutic potential in the treatment of metabolic diseases [158]. Supplementation of NGPs with their specific probiotic-targeted diets will help the strains compete with harmful microbes and acquire their niche. This combination would increase the efficacy of NGPs to be used as “*living biotherapeutics*” or food nutraceuticals [153].

The concept of a synbiotic has been redefined as a mixture of living microorganisms and substrate(s) used selectively by the host microorganism(s) that confer health benefits to the host. Two types of synbiotics have been described: synergistic synbiotics, in which the substrate is designed to be used selectively by the co-administered microorganism(s), and complementary synbiotics, in which a probiotic is combined with a prebiotic and is designed to target indigenous microorganisms [159]. In a randomised controlled trial of overweight adults, they administered a synbiotic containing *Bifidobacterium animalis* subsp. *lactis* B420TM plus Litesse1 Ul-traTM polydextrose for six months and observed a correlation between changes in the microbiome, metabolite profile, waist ratio, and energy intake. Authors detected an increase in *Akkermansia* and *Chiristensenellaceae* spp., which correlated positively with lean body mass. *Bifidobacterium* was enriched in the B420 group, and a mechanism was postulated whereby this bacterium could alter host metabolism by enhancing the intestinal barrier function [160]. Another study showed that supplementation with a symbiotic containing *Lactobacillus acidophilus*, *Bifidobacterium lactis*, *Bifidobacterium longum*, *Bifidobacterium bifidum*, and fructooligosaccharides increased gut microbiota richness after three months of intervention in healthy overweight volunteers following a weight loss diet. These authors observed an increase in *Bifidobacterium* and *Lactobacillus* in the synbiotic group, which is known to be associated with positive health effects [161].

Finally, postbiotics are bacterial fragments with or without bioactive products of microbial growth that are beneficial to the host [162]. Many lactic acid bacteria (LAB) are considered probiotics, and their postbiotic compounds have additional benefits for consumer health through the modulation of gut microbiota and metabolites [163]. Postbiotics are associated with immunoregulatory actions as they stimulate the adaptive and innate immune systems, preserve the integrity of the intestinal mucosal barrier, and antagonise microorganisms with antibiotic substances, like the activities of probiotics [164,165]. A study evaluated the gut microbiota-modulating effect of potential postbiotics produced by *Lactobacillus parabuchneri* MF2103 (LPP), a food isolate [166]. The authors performed in vitro fecal fermentation of LPP with *Escherichia*-*Shigella* inoculum (group E) and *Faecalibacterium*-enterotype inoculum (group F). They found that the relative abundance of nine and twenty-nine genera changed significantly in groups E and F, respectively, and the Shannon index of group F increased. Furthermore, total short-chain fatty acids increased significantly from 8.28 ± 0.61 mmol/L in group E to 50.54 ± 3.14 mmol/L, and from 22.15 ± 5.19 mmol/L in group F to 50.37 ± 2.80 mmol/L. Group E showed upregulation of gamma-aminobutyric acid and floretin, and downregulation of indole 5-methoxyindoleacetate and succinic acid, whereas group F showed upregulation of 7,8-diaminonononanoate and S-allyl cysteine, and downregulation of N2-acetyl-l-aminoadipate and (R)-salsolinol.

### 5.4. Essential Fatty Acids

Essential fatty acids, specifically omega-3 polyunsaturated fatty acids (PUFAs), participate in the regulation of intestinal immunity and maintenance of intestinal homeostasis, which are related to gut microbiota, fatty acid metabolism, and intestinal health [167]. omega-3 PUFAs cannot be synthesised by the human body and must be obtained directly from the diet or converted from ingested alpha-linolenic acid (ALA). However, only a small fraction of ALA can convert into essential fatty acids such as eicosapentaenoic acid (EPA), docosapentaenoic acid (DPA) and docosahexaenoic acid (DHA). Because of this limitation in conversion, dietary supplements or pharmaceutical preparations containing these unsaturated fatty acids are essential to ensure adequate intake and maintain good health [168]. In a case report examining the impact of an omega-3 PUFA-rich diet on the human intestinal microbiota, a significant increase was observed in several short-chain fatty acid (SCFA) (butyrate)-producing genera. These include *Blautia*, *Bacteroides*, *Roseburia*, and *Coprococcus* [169]. Supplementation with omega-3 PUFAs induces a reversible increase in the abundance of several short-chain fatty acid (SCFA)-producing bacteria, including *Bifidobacterium*, *Roseburia*, and *Lactobacillus* in the mouse intestinal tract [170].

### 5.5. Plant-Derived Bioactive Components

Plant-derived bioactive compounds can promote host intestinal health by regulating the structure and abundance of gut microbiota and bacterial flora metabolites. Bioactive compounds, such as curcumin, capsaicin, quercetin, resveratrol, catechin, and lignans, have been extensively studied for their wide range of pharmacological and biological activities. These include anti-inflammatory, antioxidant, anti-stress, antitumor, and antiviral properties, as well as their ability to reduce blood glucose and lipid levels and enhance insulin sensitivity [171]. Curcumin is a polyphenol isolated from the rhizomes of *Zingiberaceae* and *Araceae* plants. It has been shown to reduce intestinal bacteria, such as *Prevotellaceae*, *Enterobacteriaceae*, and *Rikenellaceae*, and increase the abundance of *Bacteroides* and butyrate-producing bacteria [172]. In obese mice, curcumin can be metabolised to curcumina-O-glucuronide by the gut microbiota and can significantly increase the relative abundance of *Lactococcus*, *Parasutterella*, and *Turicibacter* [173]. It has also been shown that obese mice fed curcumin (0.2%) can change the composition of the gut microbiota by reducing the proportion of *Bacillota* and *Bacteroides*, as well as the abundance of endotoxin-producing *Desulfovibrio* bacteria. In addition, it increased the abundance of SCFA-producing bacteria, such as *Akkermansia*, *Bacteroides*, *Parabacteroides*, *Alistipes*, and *Alloprevotella* [174]. Furthermore, a human randomised placebo-controlled trial [175] investigated the effects of turmeric and curcumin dietary supplementation vs. placebo on 30 healthy subjects (10 for each group). The turmeric tablets contained 1000 mg curcuma longa plus 1.25 mg extract of piperine and the curcumin tablets contained 1000 mg curcumin plus 1.25 mg extract of piperine. The subjects were instructed to take three tablets orally with food, twice a day (total 6000 mg daily). All the subjects showed both significant variations in microbiota composition over the time and an individualised response to treatment. The responsive subjects showed uniform increases in *Clostridium* spp., *Bacteroides* spp., *Citrobacter* spp., *Cronobacter* spp., *Enterobacter* spp., *Enterococcus* spp., *Klebsiella* spp., *Parabacteroides* spp., and *Pseudomonas* spp. Furthermore, common to these subjects was the reduced relative abundance of several *Blautia* spp. and most *Ruminococcus* spp.

Resveratrol is another natural polyphenolic compound that is widely found in grapes, *Polygonum cuspidatum*, peanuts, and other plants [176]. It has been shown that resveratrol can regulate the composition of the gut microbiota and reduce the number of opportunistic pathogens in the body [177]. A study in mice showed that resveratrol supplementation can increase the relative abundance of *Lactobacillus* and *Bifidobacterium* in the gut [178]. Another investigation showed that resveratrol was able to protect the host from colitis by reversing the development of pathogenic microbiota such as *Bacteroides acidifaciens* and the depletion of beneficial bacteria such as *Rhinococcus gnavus* and *Akkermansia muciniphila* to inhibit Th1/Th17 inflammatory cells [179].

Berries are rich in phenolic compounds such as phenolic acids, flavonols, and anthocyanins. The main site of metabolisation of the complex polyphenols to smaller phenolic compounds is the gut, through the action of microorganisms, and reciprocally, polyphenols and their metabolites can also modulate the microbial populations. In healthy subjects, these modulations lead to an increase in *Bifidobacterium*, *Lactobacillus*, and *Akkermansia*, therefore suggesting a prebiotic-like effect of the berries or their compounds [180]. A double-blind, placebo-controlled study on 28 obese women with *Schisandra chinensis* fruit (SCF) or placebo was conducted for 12 weeks. An increase in *Akkermansia*, *Roseburia*, *Bacteroides*, *Prevotella*, and *Bifidobacterium* showed a greater increase in the SCF group after treatment, while *Ruminococcus* showed a greater decrease [181].

## 6. Functional Foods

Functional foods are novel foods that have been formulated so that they contain substances or live microorganisms that have a possible health-enhancing or disease-preventing value, and at a concentration that is both safe and sufficiently high to achieve the intended benefit. The added ingredients may include nutrients, dietary fibre, phytochemicals, other substances, or probiotics [182]. They are classified into three groups: conventional foods (vegetables, fruits, fish, dairy, legumes, and cereals that have health benefits), modified foods (enriched or fortified with a specific nutrient to promote health benefits, such as calcium, antioxidants, and vitamin-fortified beverages; bread fortified with calcium and folic acid; and products enriched with plant fibres, sterols, and omega-3 fatty acids), and dietary ingredients (prebiotics) [183]. Natural antioxidants are widely found in plant-based functional foods and medicinal plants. Polyphenols are the key bioactive ingredients responsible for their antioxidant properties [184]. For example, lycopene, an antioxidant present in tomatoes, has been found to reduce the ratio of *Bacillota*/*Bacteroidota*, *Rikenella*, and *Enterorhabdus*, and increase the abundance of *Lactobacillus* in mice [185].

Broccoli is another vegetable rich in polyphenols such as isorhamnetin, synaptic acid, quercetin, and rutin. Kaczmarek et al. [186] conducted a randomised, controlled, crossover feeding study in two 18-day treatment periods, separated by a 24-day washout period, in healthy adults (*n* = 18). The participants were fed a weight-maintenance diet during the intervention period, which included 200 g of cooked broccoli and 20 g of raw Daikon radish per day. Beta diversity analysis indicated that the bacterial communities were affected by the treatment (*p* = 0.03). Broccoli consumption reduced the relative abundance of *Bacillota* by 9% compared with the controls (*p* = 0.05), increased the relative abundance of *Bacteroidota* by 10% compared with the controls (*p* = 0.03), and increased the relative abundance of *Bacteroides* by 8% compared with the controls (*p* = 0.02). Furthermore, the administration of soy isoflavones (10 mg/kg/day) to five-week-old Kunming mice for 15 days showed that mice consuming soy isoflavones had a higher food intake but a lower rate of body weight gain than normal mice. *Lactobacillus*, *Adlercreutzia*, *Coprococcus*, *Ruminococcus*, *Butyricicoccus*, and *Desulfovibrio* were the differential gut bacteria among the mice treated with soy isoflavones. In addition, acetic acid, valeric acid, isobutyric acid, isovaleric acid, and caproic acid decreased, while butyric acid and propionic acid increased in mice treated with soy isoflavones [187].

Fruits are also a source of functional foods rich in fibre, vitamins, polyphenolic compounds, and anthocyanins. In a study of 5-month-old ovariectomised Sprague-Dawley rats, purified extracts of blueberry polyphenols (0, 50, 250 or 1000 mg total polyphenols/kg bw/day) or freeze-dried blueberries (50 mg total polyphenols/kg bw/day, equivalent to 250 g fresh blueberries/day in humans) were administered by gavage for 90 days. The intestinal microbial populations showed increased diversity at moderate doses, but decreased diversity at high doses. After 90 days of treatment, cranberry polyphenols induced a significant dose-dependent change in the microbiota community structure (*p* < 0.01), with the mean ratio of *Bacillota* to *Bacteroides* decreasing from 5.4 (water control group) to 2.2 (medium group) and 0.7 (high group). Additionally, *Pseudomonadota* increased in the high-dose group [188]. *Punica granatum* L., commonly known as pomegranate, is an abundant source of polyphenols, including hydrolysable ellagitannins, ellagic acid, anthocyanins, and other bioactive phytochemicals that have been shown to be effective in defending against oxidative stress and have immunomodulatory activities. No significant changes in gut microbial diversity were observed in either cohort after four weeks of intervention, but there was a significant increase in the relative abundance of *Coprococcus eutectus*, *Roseburia faecis*, *Roseburia inullnivorans*, *Ruminococcus bicirculans*, *Ruminococcus calidus*, and *Faecalibacterium prausnitzii*. Pomegranate extract supplementation resulted in an increase in circulating propionate levels (*p* = 0.02) and a trend towards increased acetate levels (*p* = 0.12) [189].

Nuts are also functional foods rich in fibre and polyphenols, both of which are used as substrates by gut microbiota. García-Mantrana et al. [190] examined the effects of 33 g walnuts per day for 3 days on intestinal microbiota. Walnut intake significantly decreased the relative abundance of *Bacteroidota*, while increasing the abundance of *Actinomycetota*. Participants classified as UM-A showed a significant decrease in the *Lachnospiraceae* family, as well as a significant increase in the genera *Coprococcus* and *Collinsela*. Participants classified as UM-B showed a significant increase in the family *Coriobacteriaceae* as well as significant increases in the genera *Coprococcus*, *Collinsella*, *Bifidobacterium*, and *Blautia*. Another study evaluated the effect of ingesting 57 g of roasted almonds on the gut microbiota for 6 weeks in a control group receiving graham crackers. When considering α-diversity, almond feeding resulted in significant increases in the Chao-1 index, observed out, and Shannon index, whereas Simpson’s index decreased significantly. Similarly, significant differences in β-diversity between the treatment groups were identified according to the unweighted UniFrac distance and Bray–Curtis’s dissimilarity metric. At the genus level, they observed a significant decrease in *Alistipes*, *Butyricimonas*, and *Odoribacter*, and a significant increase in the genus *Lachnospira*. At the species level, there was a 48% decrease in the relative abundance of *Bacteroides fragilis* [191].

## 7. Alternative Foods

A randomised controlled study found that occasional replacement of animal meat with plant-based products (PBMAs) such as burgers, sausages, and meatballs can promote positive changes in the gut microbiota of consumers. In the intervention group that consumed PBMAs, an increase in butyrate production potential was observed through the 4-aminobutyrate/succinate and glutamate pathways, as well as an increase in the conjoint abundance of butyrate-producing taxa compared to the control group. A decrease in the phylum *Mycoplasmatota* was observed in the intervention group and increased in the control group. These findings suggest that occasional replacement of animal meat with PBMAs in flexitarian dietary patterns may have beneficial effects on gut microbiota [192]. These authors concluded that PBMAs manufactured with plant ingredients rich in protein, fibre, and phenolic compounds can induce positive changes in the gut microbiota, even if their consumption is only occasional.

Marine algae are rich in bioactive ingredients with diverse biological activities and potential health benefits [193]. A study on hamsters showed that hamsters fed a diet rich in fat and sucrose when consuming edible algal oligosaccharides showed an increase in the *Bacteroidota* population and a decrease in plasma glycemia [194]. Li et al. [195] showed that after an unsaturated alginate oligosaccharide (UAOS) treatment, the concentration and variety of the gut microbiota increased, represented by *Akkermancia* spp., *Lactobacillus* spp., *Bifidobacterium* spp., and *Saccharomyces* spp., with the potential of lowering body weight gain and improving glucose and lipid homeostasis. High-fat diet (HFD)-induced obesity markedly altered the percentage of gut microbiota phyla, while UAOS treatment significantly reversed this tendency, suggesting that UAOSs can regulate the gut microbiota. *Durvillaea antarctica* is another seaweed whose composition is notable for its contribution to glucans and polysaccharides such as fucoidan, laminarin, alginate, ulvan, and porfirian, which are unique to marine algae [196]. These dietary components have been reported to have biological activity associated with anti-cancer, antidiabetic, and anti-inflammatory functions, as well as immunomodulatory effects. The polysaccharide alginate, extracted from *D. antarctica*, has been reported to play a crucial role in intestinal health. It promotes the growth of beneficial *Bifidobacteria* and inhibits pathogenic bacteria, which prolongs colonic fermentation [196].

Researchers have also investigated how edible insects can alter the composition of human gut microbiota. Young et al. [197] used an in vitro model to simulate oral, gastric, and intestinal digestion followed by fecal fermentation to analyse the effect of different insects on the microbiota. The results showed that each type of insect caused significant differences in the composition of the microbiota compared to the controls. For example, grass cricket larvae increased the proportion of *Faecalibacterium*, whereas black field crickets increased the prevalence of the *Escherichia/Shigella* group and *Dialister*. Wax moth larvae promoted the growth of an unclassified *Lachnospiraceae* group and the *Escherichia/Shigella* group.

Mushrooms are abundant in polysaccharides, proteins, vitamins, minerals, trace elements, and antioxidants [198]. The diverse bioactive compounds in mushrooms, such as polysaccharides, proteins, polyphenols, alkaloids, steroids, and terpenes, contribute to their beneficial health effects [199]. For instance, soluble dietary fibres from *Lentinula edodes* have been shown to enhance intestinal fermentation, increase the concentration of short-chain fatty acids (SCFAs), such as propionic and butyric acid, and boost the number of *Bacteroides* species [200]. Additionally, an in vitro study revealed that β-glucans found in species such as *Pleurotus ostreatus*, *Pleurotus eryngii*, and *Hericium erinaceus* function as prebiotics, particularly in the elderly population. These β-glucans increase the cell count of *Lactobacillus* spp. strains and stimulate the synthesis of SCFAs, especially propionate and butyrate [201].

Fermented products such as kimchi and kefir are well-known for their positive effects on the gut microbiome [41,200,201,202]. However, newer fermented products, like tempeh made from various legumes and kombucha from different bases, are explored for their additional benefits. In an uncontrolled open-label study involving ten healthy human volunteers, the consumption of tempeh increased the abundance of *Akkermansia muciniphila* in feces and the concentrations of immunoglobulin A [203]. Another pilot study employed a randomised crossover design with two two-week intervention phases separated by a two-week washout period. Six healthy male volunteers consumed 150 g/day of various fermented foods, including traditional sauerkraut, fermented beetroot, ginger-carrot, turmeric cauliflower, daikon kimchi, and pink sauerkraut. Each product was assigned to be consumed on a specific day of the week, with traditional sauerkraut consumed twice weekly. Following the consumption of these foods, a slight increase in the alpha diversity of the gut microbiota was observed. Specifically, the genus *Prevotella* decreased, whereas *Bacteroides* increased after both the intervention phases [204].

## 8. Diet

The health benefits of the Mediterranean diet (MD), including its beneficial effects on the gut microbiome, have been recognised [205] as it is rich in fruits, vegetables, legumes, nuts, and olive oil. De Filippis et al. [206] showed that a Mediterranean diet, characterised by a high intake of plant foods, has a beneficial effect on the composition of the gut microbiota. Subjects who consume a higher proportion of plant foods have a higher proportion of SCFAs and fibre-degrading bacteria in their feces. In contrast, subjects with poor adherence to the MD had a higher concentration of trimethylamine N-oxide in their urine. In a subsequent study, Mitsou et al. [207] confirmed the positive effects of the MD on the gut microbiota profile. In fact, subjects with higher adherence to the MD, as monitored with the MedDietScore, had a lower presence of *E. coli*, an increased presence of total bacteria, a higher ratio of *Bifidobacterium*/*E. coli*, and an increased prevalence of *Candida albicans* and SCFAs. Garcia-Mantrana et al. [208] pointed out that a higher adherence to the MD is characterised by an increase in *Bifidobacterium* and a higher percentage of SCFAs. Therefore, the MD has a positive influence on the gut microbiota, specifically on its diversity and metabolic activity. A recent cross-sectional study investigated gut microbial composition and its association with the MD in a sample of healthy young Italian adults. At the genus level, *Streptococcus* and *Dorea* were highly abundant in overweight/obese individuals, *Ruminococcus* and *Oscillospira* in participants with lower adherence to MD, and *Lachnobacterium* in subjects with low physical activity (*p* = 0.001). A significantly higher abundance of *Prevotella* was found in individuals with lower BMIs, lower adherence to the MD and lower physical activity levels (*p* = 0.001) [209].

The strictest form of vegetarianism, known as veganism, involves the complete exclusion of all meat and animal-derived products, including dairy, eggs, and honey [208]. Vegans adhere to a diet exclusively composed of plant-based foods, such as grains, vegetables, fruits, legumes, nuts, seeds, and plant oils and fats [210]. The gut microbiota is dominated by *Prevotella* species in individuals with plant-based dietary habits, such as populations living in African, Asian, and South American societies, while *Bacteroides*-driven enterotypes are predominant in individuals living in Western societies that consume diets rich in animal protein, amino acids, and saturated fats [211,212]. A previous study compared the gut microbiota of people following a vegan diet with that of omnivores. Vegans showed greater bacterial diversity and a higher abundance of beneficial bacteria, such as *Prevotella* and *Bifidobacterium*. In addition, this diet was associated with lower levels of trimethylamine N-oxide, suggesting that a vegan diet may contribute to better cardiovascular and metabolic health through changes in the microbiome [213]. Another study investigated the specific impact of vegan, vegetarian, and omnivorous diet types on the gut microbiota composition of 101 adults among homogeneous groups for variables such as age, anthropometric variables, ethnicity, and geographic area. Vegetarians were significantly richer than omnivores were. Counts of *Bacteroidota*-related operational taxonomic units (OTUs) were higher in vegans and vegetarians than in omnivores [214].

The ketogenic diet (KD) is a dietary regime focused on strongly reducing carbohydrate intake and increasing fat intake, leading to a state of ketosis [215]. Different protocols exist in ketogenic diets that differ in caloric content and macronutrient percentage, which have applications in neurological and metabolic disorders [216]. Ang et al. [215] examined the effects of a ketogenic diet on 17 non-diabetic overweight or obese class I adult men. Participants were first fed a baseline diet for 4 weeks (50% carbohydrate, 15% protein, 35% fat), followed by a 4-week ketogenic diet (5% carbohydrate, 15% protein, 80% fat), resulting in an increase in circulating plasma ketone bodies. Significant changes in the gut microbiota were observed between the two diets, including an altered relative abundance of *Actinomycetota*, *Bacteroidota*, and *Bacillota*. Nineteen genera were significantly different, with *Bifidobacterium* showing the greatest decrease in the KD. This decrease was mediated by the increased production of ketone bodies, most importantly beta-hydroxybutyrate, and led to lower levels of intestinal and visceral fat proinflammatory Th17 cells. Moreover, a randomised pilot study compared the addition of whey, vegetables, or animal protein to a 45-day very low-calorie ketogenic diet (40.4% fat, 46.1% protein, and 13.5% carbohydrate) in 48 obese Italian adults and observed weight loss in all groups [215,217]. The study also showed that all very low-calorie ketogenic diets resulted in a healthier composition of the microbiota due to a decrease in *Bacillota* and an increase in *Bacteroidota*, which was more pronounced in the whey and vegetable protein groups.

## 9. New Eating Habits

Intermittent fasting (IF) has gained popularity as an intervention for overweight, obesity, and metabolic syndromes. Fasting refers to abstention from consuming food and/or drinks for various periods [218]. Fasting can be classified as intermittent or prolonged [219]. The most common forms include time-restricted feeding (TRF), where the fasting period occurs within 24 h and typically lasts between 12 and 18 h per day. Other forms of IF include alternate-day fasting (ADF), which involves alternating days of *ad libitum* eating with fasting. The 5:2 diet is a modified version of ADF, where fasting occurs on two specific days of the week and the remaining five days involve *ad libitum* eating. Intermittent fasting can influence the composition of the gut microbiome, thereby affecting various microbiome-mediated functions in humans. A human intervention study compared the effects of TRF with those of a non-restricted feeding (non-TRF) control group. Parameters related to metabolism, liver function, inflammation, and the gut microbiota were evaluated. TRF is associated with increased diversity of the gut microbiota. Specifically, TRF enriched two bacterial families in the gut microbiota, *Prevotellaceae* and *Bacteroidaceae*. An association was observed between gut microbiome richness and sirtuin-1 (Sirt1) expression, as well as HDL cholesterol levels. There was a negative correlation between the abundance of the *Bacteroidia* class and levels of LDL cholesterol and triglycerides. Additionally, an increase in circadian gene expression was noted, through the activation of Sirt1, which is associated with gut microbiota richness. These changes in gut microbiota may contribute to the beneficial effects of TRF on metabolic health and circadian rhythms [220].

In addition, personalised diets, which rely on genetic, metabolic, and microbiome data to design a specific dietary plan, are gaining attention for their potential to positively influence the human gut microbiome. Zeevi et al. [221] investigated the postprandial glycemic response (PPGR) to different meals and the factors influencing this response. The study recruited 800 individuals aged 18-70 years who had not previously been diagnosed with type II diabetes. Participants were connected to a continuous glucose monitor (CGM) for 7 days and recorded their activities and food intake in real time via a website adapted to their smartphone. This study also developed a machine learning algorithm that integrated clinical and microbiome factors to predict personalised PPGRs. The algorithm achieved high accuracy in predicting PPGRs and was validated in an independent cohort of 100 participants. In addition, a randomised controlled trial was conducted to evaluate the efficacy of personalised dietary interventions based on the algorithm’s predictions. The results showed that the interventions significantly improved PPGR and induced changes in the gut microbiota. Overall, the study demonstrated the variability of PPGRs, the associations between PPGRs and risk factors, and the potential for personalised dietary interventions to improve PPGRs. The study also explored associations between microbiome characteristics and PPGR variability, identifying possible metabolic pathways and pathways that may mediate these effects. This personalised approach to predicting PPGR to food was recently validated in a non-diabetic population in the United States [222]. Recently, a large-scale twin study revealed high interpersonal variability in postprandial responses (glycemic, insulinemic, and lipemic responses) to diets, highlighting that even genetically similar twins respond differently to identical meals [223]. These results support the idea that, to achieve the same outcome in different individuals, personalised dietary approaches are necessary. However, such a “tailored nutritional approach” requires the development of more feasible and sustainable personalised nutritional strategies to optimise the gut microbiome and improve host responsiveness [224].

## 10. Effects of Novel Foods on the Human Gut Microbiome

The incorporation of novel foods into the daily diet has generated considerable interest due to their potential benefits for gut health. A classification of novel foods is presented in Table 3.

Foods rich in bioactive compounds such as polyphenols promote the growth of beneficial bacteria such as *Bacillota*, *Lactobacillus*, and *Bifidobacterium* [239]. Probiotics are emerging as innovative strategies to modulate gut bacterial communities and combat food allergies, highlighting the intricate relationship between novel foods and the gut microbiome [240]. For example, recent studies have shown consumption of edible insects, a category of novel foods [241]. Along with increasing novel food applications, edible fungi have been considered as potential sources of biological nutraceuticals [242]. Likewise, processed meat products enriched with prebiotics in animal studies have shown an increase in *Bacillota* and a decrease in *Bacteroidota* at the phylum level. At the genus level, *Ruminococcaceae*, *Staphylococcus*, and *Bifidobacterium* were increased in rats in the group fed both calcium-rich and inulin-enriched sausages [243]. A series of studies on novel foods on the gut microbiome are listed in Table 4.

## 11. Critical Analysis and Future Directions

Novel foods include innovative technologies and production processes, as well as new sources of agricultural products and ingredients from microorganisms, fungi, algae, and insects [263]. Furthermore, the incorporation of novel raw materials and emerging technologies enables the extension of food shelf life. These foods have been shown to be important sources of bioactive compounds such as polyphenols, proteins, dietary fibre, and essential fatty acids. These findings indicate that novel foods not only represent sustainable and nutritious alternatives to traditional foods but can also play a crucial role in promoting the gut microbiome. For example, the consumption of seaweed has shown prebiotic effects, stimulated the growth of beneficial bacteria, and reduced pathogenic bacteria, which is promising for gut health. However, variability in the composition of algae according to their geographical origin and cultivation methods poses challenges in terms of standardisation and food safety.

In addition, new-generation fermented products such as tempeh made from various legumes have shown to increase the abundance of beneficial bacteria such as *Akkermansia muciniphila*, which is associated with metabolic and anti-inflammatory benefits. However, the complexity of the fermentation processes and the need to maintain optimal production conditions underline the importance of strict regulations and quality control. Although preliminary studies have highlighted their positive effects on the gut microbiome and overall health, considerable challenges remain to be overcome. For example, cultural acceptance and psychological barriers to certain novel foods, such as edible insects, may limit their widespread adoption, despite their nutritional and environmental benefits. In addition, scientific evidence of the long-term effects of these foods on human health is limited and requires larger and longer studies to draw definitive conclusions.

Cultured meat represents another innovation in novel foods, promising a reduction in resource use and greenhouse gas emissions compared to traditional livestock farming. Despite its environmental and ethical benefits, its excessive cost of production and the lack of long-term data on its effects on human health remain critical barriers. Additionally, public perception and acceptance of these viable alternatives are still under study. Although novel foods offer promising nutritional and sustainable solutions to improve human health through the modulation of the gut microbiome, more research is needed on the interaction between the nutrients in these foods and the microbiome, as well as their ability to promote microbial diversity and abundance. In addition, it is important to consider the regulation of these foods and their assessment in terms of quality and safety to minimise potential risks. Moreover, the cultural challenges associated with their adoption and consumption must be addressed. Continued research, public education, and the development of robust policies are critical to effectively integrate these foods into people’s diets and promote their health and environmental benefits.

## 12. Conclusions

There is growing evidence in the scientific literature that novel foods have positive effects on host health through interactions with the gut microbiome. Different studies mentioned in this review, both in vitro and in vivo, demonstrate the relationship between nutrient content, nutritional supplements, diet type, eating habits, and the gut microbiome. These interactions selectively promote specific microbial species and increase microbiome diversity, which has been associated with a reduced risk of chronic diseases. However, a clear and in-depth understanding of the mechanisms underlying the health-promoting properties of novel foods and their role in maintaining the diversity and functionality of the gut microbiome to prevent the onset and progression of chronic diseases is still lacking, highlighting the need for further research in this area.

## Figures and Tables

**Table 1:** Novel food definitions *.

Region/Country	Definition	Reference
European Union	Food produced using recent technologies and production processes or that is or has been traditionally consumed outside the EU and has not been consumed in the EU to a significant degree.	[19]
United Kingdom of Great Britain and Northern Ireland	A food that has not been significantly consumed by humans in the UK before 15 May 1997 is classified as a “novel food” under UK legislation.	[20]
India	A food may lack a history of human consumption, or its ingredients and sources may not have been previously consumed. Alternatively, it could be a food or ingredient produced using recent technology or innovative engineering processes. These methods may alter the size, composition, or structure of the food or its ingredients, potentially affecting its nutritional value, metabolism, or the presence of undesirable substances.	[21]
Israel	A newly developed, innovative food created through modern technologies and production methods, or a food traditionally consumed outside of Israel.	[22]
Gulf Cooperation Council	Novel food is composed of, isolated from, or produced through cell culture or tissue culture derived from animals, plants, microorganisms, fungi, or algae.	[23]
China	Any food not traditionally consumed in China, including organisms, extracts from organisms, food ingredients with modified structures, or newly developed food components.	[24]
Japan	Foods with no history of safe use or those produced using new processes must undergo a novel food safety assessment.	[25]
Singapore	Foods that have not been widely consumed as food for at least 20 years, whether within Singapore or internationally.	[26]
Thailand	Any substance used as food or a food ingredient that has been widely consumed by humans for less than 15 years based on scientific or reliable evidence, or that has undergone a production process not currently in use, where this process significantly alters the composition or structure of the food, impacting its nutritional value, metabolism, or the presence of undesirable substances.	[27]
Republic of Korea	Food ingredients that have no history of consumption in Korea.	[28]
Australia and Nueva Zelanda	Novel foods, which are not traditionally consumed, must be evaluated by Food Standards Australia New Zealand to ensure their safety before they can be introduced into the food supply.	[29]
United States of America	There is no explicit definition for novel foods; however, any new food substance must receive premarket approval from the US FDA unless it is classified as Generally Recognized as Safe (GRAS).	[30]
Canada	Novel foods fall into three major categories: Substances with no history of safe use as food. Foods manufactured, prepared, preserved, or packaged using new processes. Foods derived from plants, animals, or microorganisms that have been genetically modified.	[31]
Brazil	Novel foods are substances with no history of consumption in Brazil, or foods containing substances that are already consumed but at levels significantly higher than those currently observed in regular diets. These foods require premarket approval, which may be renewed every five years.	[32]

* To date, these are the only regions and countries that have defined novel foods.

**Table 2:** Definition of microbiome.

Definition	Key Issues	Author	Reference
Communities of symbiotic and pathogenic microorganisms that share the human body space.	Introduces the coexistence of symbionts and pathogenic microorganisms that cohabit in the human body.	Lederberg and McCray	[1]
Includes all microorganisms and their genetic material in a human organism as well as the environmental conditions that affect them.	The influence on host physiology and health focuses on a specific environment.	Duncan et al.,	[53]
Collection of microorganisms that live in various habitats of the human body, each with distinct functions that affect health and disease.	Diversity of habitats within the human body and their distinct functions.	Turnbaugh et al.	[54]
Set of genes harbouring the microbial communities that coexist with humans in the gut.	Variability in the composition of the microbiome between individuals and over time within a single person.	Ursell et al.	[55]
A complete habitat includes microorganisms (bacteria, archaea, lower and higher eukaryotes, and viruses), their genomes (genes), and the surrounding environmental conditions.	They encompass both biotic and abiotic factors in specific environments.	Marchesi and Ravel	[56]
The human microbiome encompasses all microorganisms residing in the human body, estimated at around 39 trillion bacteria, which surpasses the number of human cells.	Specific number of bacteria compared to human cells.	Sender, Fuchs, and Milo	[57]
This concept applies not only to bacteria-mediated conditions in the gut but also to viral, fungal, and host–microbe interactions throughout the human body. This includes environmental microbial communities that interact with human health, such as those in the built environment, livestock, agriculture, and pets.	Potential in public health to discover new biomarkers, therapeutics, and molecular mechanisms in human populations.	Wilkinson et al.	[51]
The microbiome is a community of microorganisms, including bacteria, fungi, archaea, protists, and viruses, whose genetic material it found within or on a specific host organism. These microorganisms interact with each other and their environment to create distinct ecological niches. Microbiomes evolve over time and across different scales, and are intricately connected with macro-ecosystems that include eukaryotic hosts, where they play a vital role in overall functioning and health.	It covers the diversity of microorganisms, their functions, and their interactions within a habitat with different physicochemical properties.	Berg et al.	[2]
Interactive and fluctuating communities of microbes colonise and develop on surfaces, including those associated with host organisms.	Vertical transmission refers to the transfer of microorganisms from mother to child during pregnancy, childbirth, and breastfeeding.	Murphy et al.	[52]
A complete community of microorganisms inhabits a given environment, such as the human body, animals, or the environment.	The holistic concept in the field of One Health facilitates interactions between humans, animals, and the environment, along with co-evolution, co-development, comet metabolism, and co-regulation with humans and animal partners.	Ma et al.	[50]
Collection of dynamic microbial communities inhabiting diverse anatomical locations in the human body. These microbial communities co-evolve with the host and play key roles in promoting human health.	A diverse array of microorganisms, including bacteria, archaea, fungi, viruses, and mobile genetic elements, have been identified. These microorganisms influence host physiology and play a crucial role in metabolism and immune system development.	Aggarwal	[11]

**Table 3:** Classification of novel food.

Classification	Type	Description	Reference
Foods and food ingredients derived from new production process	Cell-based food production	In vitro cultivation of animal cells followed by processing into products that resemble conventionally sourced meat.	[225]
	Nanotechnology in food	Use of nanotechnology to improve the texture, taste, preservation, and nutritional properties of foods.	[226]
	UV-treated food	Exposure of food to UV-C radiation, which has a wavelength of between 200 and 280 nanometres.	[227]
	Precision fermentation	Use of genetically engineered microorganisms to produce food substances in a bioreactor, usually using simple starting materials such as sugar and glycerol.	[228]
	Vertical farming	New modality in indoor farming that has gained popularity in recent years due to increasing urbanisation coupled with food security concerns.	[229]
	3D printing of foods	The use of a computer-controlled robotic process to construct solid food layers and fusing these layers together using physical or chemical methods.	[230]
Foods and food ingredients with a new or intentionally modified primary molecular structure	Genetically modified organisms (GMOs)	Foods that have been genetically altered to enhance their performance, resistance, or nutritional value.	[231]
Foods and food ingredients consisting of or isolated from microorganisms, fungi, or algae	Microbial protein	Proteins obtained from bacteria, fungi, and microalgae.	[232]
	Seaweed and microalgae	Seaweed and microalgae used as a source of protein, natural omega-3 long-chain fatty acids, soluble dietary fibres, vitamins, and minerals.	[233]
Foods and food ingredients consisting of or isolated from plants or their parts	Plant-derived proteins	Derived from plant materials through chemical and mechanical processing, which effectively removes carbohydrates, lipids, and other non-protein components, resulting in a mixture where protein is the primary component.	[234]
	Sugar substitutes	Ingredients or products that replace traditional food components.	[235]
	Plant-based alternatives	Primarily consuming plant-based foods, including fruits, vegetables, nuts, seeds, legumes, and whole grains.	[236]
New food ingredients isolated from animals	Insects	Edible insects used as a source of protein and other essential nutrients.	[237]
	Jellyfish	Edible species typically have low carbohydrate and lipid content, high protein levels (primarily collagen), and different essential minerals.	[238]

**Table 4:** Potential food ingredients that can be classified as novel foods.

Food	Effect in Gut Microbiome	Type of Study	Reference
Bamboo (insoluble dietary fibre)	Increases *Bifidobacterium adolescentis* and *Lactobacillus acidophilus*	In vitro	[244]
Inulin supplementation Inulin-propionate ester supplementation	Increase *Actinomycetota* and decrease *Clostridia* with inulin supplementation. Higher proportion of *Anaerostipes hadrus*, *Bifidobacterium faecale*, and *Bacteroides caccae*. Lower proportion of *Blautia obeum*, *Blautia luti*, *Oscillibacter* spp., *Blautia faecis*, and *Ruminococcus faecis*. Higher proportion of *Bacteroides uniformis*, *Bacteroides xylanisolvens*, and *Fusicatenibacter saccharivorans*. Lower proportion of *Blautia obeum*, *Anaerostipes hadrus*, *Bifidobacterium faecale*, *Prevotella copri*, and *Eubacterium ruminantium*	In vivo	[245]
Powder from crickets *Acheta domestica*, silkworm pupae *Bombyx mori*, isolated chitin	Fluctuations in the relative abundance of *Pseudomonadota*, *Bacteroidota*, and *Actinomycetota* and the families *Bifidobacteriaceae*, *Prevotellaceae*, and *Lachnospiraceae*.	In vitro	[246]
	Chitin induces the growth of *Ruminococcaceae*, and *Lachnospiraceae*, *Faecalibacterium*, and *Roseburia*		
Two *Laminaria japonica* polysaccharides	Fraction with lower molecular weight is better at promoting the proliferation of *Akkermansiaceae.* Fraction with higher molecular weight reduces the *Bacillota/Bacteroidota* ratio and increases the content of *Bacteroidaceae* and *Tannerellaceae*	In vitro	[247]
Red seaweed (*Gracilaria fisheri*)	Increases the diversity of *Roseburia* and *Faecalibacterium*	In vitro	[248]
*Moringa oleifera*	Increases the abundance of *Bacteroidota* and decreases the abundance of *Pseudomonadota*	In vitro	[249]
Cricket flour (prebiotic)	*Bacteroidota* and *Bacillota* make up ~90% of sequences at phylum level.Increases *Actinomycetota* (*Bifidobacterium animalis*), decreases *Lactobacillus reuteri*, *Leuconostoc*, and *Acidaminococcus* genus	In vivo	[250]
Flaxseed supplementation	Decreases the abundance of *Bacillota*, *Pseudomonadota*, *Bacteroidota*	In vitro	[251]
Novel probiotic preparation	Most differentially abundant genera (87%) were identified in volunteers who consumed probiotics, including *Coprococcus*, *Streptococcus*, and *Lactococcus*	In vivo	[252]
Two novel probiotic mixtures (Consti-Biome and Sensi-Biome)	Consti-Biome and Sensi-Biome compete with *Staphylococcus aureus* and *Escherichia coli*, and prevent them from adhering to HT-29 cells	In vitro	[253]
Lacto-fermented vegetables	Increase *Leuconostoc mesenteroides*, *Parabacteroides distasonis*, *Lachnospira*, *Ruminococcaceae*, *Coproccocus*, *Blautia*, *Clostridiales*, *Coprobacillus*, *AlphaPseudomonadota*, *Rhodotorula mucilaginosa*, *Penicillium*, *Starmerella*, *Cryptococcus laurentii*, *Vishniacozyma carnescens*	In vivo	[254]
Chia mucilage	Increases *Enterococcus* spp. and *Lactobacillus* spp.	In vitro	[255]
Walnut	Increases the relative abundance of *Roseburia*, *Eubacterium eligens* group, *Lachnospiraceae*, and *Leuconostocaceae*	In vivo	[256]
Noni *(Morinda citrifolia L.)* fruit	Increases *Enterobacteria*, *BetaPseudomonadota*, *Verrucomicrobiota*, and reduces Gram-positive bacteria	In vivo	[257]
Plant-based OsomeFood Clean Label meal (fungi, algae, nuts, and plant protein)	Increases *Faecalibacterium prausnitzii*, *Prevotella* CAG 5226, *Roseburia hominis*, *Roseburia* sp. CAG 182	In vivo	[107]
Studies in murine models with projection in humans			
Bamboo (insoluble dietary fibre)	Increases *Streptomyces* and *Bacteroides*. Decreases the abundance of *Alcaligenes* and *Pasteurella*	In vivo	[244]
Crude polysaccharide from *Dictyopteris divaricata* seaweed	Increases the population of *Bacillota*, *Bacteroidota*, and *Lactobacillus*	In vivo	[258]
Food supplement of *Arthrospira platensis*	*Clostridium* XIVa, *Barnesiella*, *Desulfovribrio*, and *Eubacterium* show significant differences in abundance across the treatment groups	In vivo	[259]
Edible mushroom (*Flammulina velu* polysaccharides)	Elevates the relative abundance of *Bacteroidota* and reduce the relative abundance of *Bacillota*	In vivo	[260]
Edible mushroom (*Ganoderma lucidum* and *Poria cocos* polysaccharides)	The abundance of *Porphyromonadaceae* decreases in the group treated with *G. lucidum* polysaccharides. *Eubacteriaceae* family increases in the groups treated with *G. lucidum* and *P. cocos* polysaccharides	In vivo	[261]
*Bifidobacterium longum* 070103 fermented milk	Increase in *Bacillota*, *Bacteroides*, and *Actinomycetes*. The main microbiota consists of *norank_f_Muribaculaceae*, *Allobaculum*, *Lactobacillus*, *Dubosiella*, *Faecalibaculum*, and *Bifidobacterium*	In vivo	[262]

## Data Availability

The original contributions presented in the study are included in the article, further inquiries can be directed to the corresponding author.
